# Effect of Admixtures on Durability and Physical-Mechanical Properties of Alkali-Activated Materials

**DOI:** 10.3390/ma15062010

**Published:** 2022-03-08

**Authors:** Lukáš Procházka, Jana Boháčová, Barbara Vojvodíková

**Affiliations:** Faculty of Civil Engineering, VSB-Technical University of Ostrava, L. Podeste 1875, 70800 Ostrava, Czech Republic; jana.bohacova@vsb.cz (J.B.); barbara.vojvodikova@vsb.cz (B.V.)

**Keywords:** ground granulated blast furnace slag, fly ash, cement by-pass dust, alkali-activated materials, material engineering

## Abstract

The results of ground granulated blast furnace slag (GGBS) tests in alkali-activated systems show that, with its use, it is possible to produce promising materials with the required properties. Unfortunately, GGBS is becoming a scarce commodity on the market, so the effort is to partially replace its volume in these materials with other secondary materials, while maintaining the original properties. This paper focuses on a comparison of two basic types of mixtures. The first mixture was prepared only from ground granulated blast furnace slag (GGBS) and the second type of mixture was prepared with admixtures, where the admixtures formed a total of 30% (15% of the replacement was fly ash after denitrification—FA, and 15% of the replacement was cement by-pass dust—CBPD). These mixtures were prepared with varying amounts of activator and tested. The experiment monitored the development of strength over time and the influence of different types of aggressive environments on the strength characteristics. Thermal analysis and FTIR were used in the experiment to determine the degradation products. The paper provides an interesting comparison of the resistance results of different composites in aggressive environments and at the same time an evaluation of the behavior of individual mixtures in different types of aggressive environment. After 28 days of maturation, the highest strengths were obtained with mixtures with the lowest doses of activator. The difference in these compressive strengths was around 25% in favor of the mixtures with only GGBS; in the case of flexural strength, this difference was around 23%. The largest decreases in strength were achieved in the XA3 environment. This environment contains the highest concentration of sulfate ions according to the EN 206-1 standard. The decreases in compressive strength were 40–45%, compared to the same old reference series. The surface degraded due to sulfate ions. Calcium sulphate dihydrate was identified by FTIR, thermal analysis and SEM as a degradation product.

## 1. Introduction

The environmental impacts of the cement industry need to be viewed from different perspectives. They can be divided simply into problems associated with limestone and clinker extraction and problems associated with cement production. As the global demand for cement [1] increases, the burden caused by mining is gradually increasing. Despite more efficient filtration during firing, pollution has increased with increasing volume globally [2].

The resistance of concrete to sulphates is one of the most fundamental properties that affects the durability of concrete [3,4]. The mechanism that leads to degradation of properties involves a set of physical and chemical reactions. External or internal sulphate ions in concrete react with the hydration products of Portland cement; these reactions result in expansion and structural failure, which can lead to the decomposition of concrete elements [3,4,5,6]. Sulphate ions attack calcium hydroxide and calcium aluminates, leading to the formation of gypsum and ettringite [4,7,8].

Magnesium sulphate attacks all hydration products, including CSH gel, forming brucite, gypsum and ettringite [4].

The resistance of alkali-activated materials against the action of sodium sulphate was higher than against magnesium sulphate in a similar concentration [9,10]. The main hydration product C-A-S-H gel underwent extensive decalcification and silicate polymerization when deposited in magnesium sulfate. These reactions indicate transformation to magnesium and aluminosilicate hydrate M-A-S-H and/or silica gels. Conversely, when alkali-activated materials were exposed to sodium sulfate, the structure was almost unchanged, with a low content of gypsum and etringite. Such a significant difference is associated with the types of cations in sulfate salts and their ability to change the pH in the pore solution of alkali-activated materials [9,11].

Many studies have found that alkali-activated materials have better acid resistance in comparison to ordinary Portland cement (OPC). This resistance depends on a number of physical and chemical factors, as well as on the type of acid, concentration and composition of the alkali-activated material [12,13,14,15]. Significant resistance to acids is usually attributed to the formation of decalcified and modified aluminosilicate gel after acid treatment [12,16,17]. The deteriorating properties of alkali-activated materials exposed to strong acids are caused by an initial exchange between alkaline cations Na^+^, K^+^, Ca^2+^, Mg^2+^) and hydronium ions (H_3_O^+^). As a result of this exchange, the aluminosilicate framework is destabilized, resulting in electrophilic attack on the Si-O-Al bond and the formation of a bond between the Si-OH and Al-OH groups. The Si-OH compound is believed to be amorphous silica and has some beneficial properties, so the formation of Al-OH is caused by the dealumination of the aluminosilicate gel [12].

The durability against carbonation of materials based on alkali-activated materials and traditional Portland cement is affected by water absorption and matrix permeability. The mechanism of damage of these materials is different [2,18]. It has been found that, due to the absence of calcium hydroxide in alkali-activated materials, the carbonation process occurs faster than in traditional Portland cement [18,19]. During the carbonation process of alkali-activated materials with a high Ca content, carbon dioxide dissolves in the pore solutions to form carbonic acid. Subsequently, it reacts with the C-A-S-H gel to form calcium carbonate [2,20]. Materials with a low Ca content do not decalcify, but the carbonation process of the N-A-S-H gel is associated with conversion of the pore solution from high alkalinity to a high concentration of sodium carbonate [2,21,22,23,24].

Several studies have already been carried out on the resistance of alkali-activated materials to aggressive environments, such as acids (e.g., phosphoric, sulfuric, nitric, hydrochloric and acetic) or sulphates (e.g., sodium sulphate and magnesium sulphate). Different concentrations and exposures in aggressive environments were used in these studies.

In ref. [25], the authors experimentally investigated the resistance of alkali-activated slag/fly ash-based pastes and compared them to ordinary Portland cement-based pastes exposed to phosphoric acid with different pH values for a period of 150 days. The results revealed that alkali-activated slag/fly ash pastes were more resistant to phosphoric acid compared to OPC-based pastes. They also observed that the kinetics of degradation of the pastes was different and depended on the binder. The degradation of alkali-activated slag/fly ash-based pastes was divided into an early stage and a later stage. A theoretical model was developed that predicted that alkali-activated slag/fly ash-based pastes could potentially reduce the degradation depth by around 70–80% in comparison to OPC-based pastes.

In ref. [26], the microstructure and composition evolution of two gels exposed to sulfuric acid were investigated. (N-A-S-H) and (C-(N)-A-S-H) gels were chemically prepared by laboratory synthesized aluminosilicate powders. The results showed that the molecular framework changes of N-A-S-H gel, caused by dealumination due to sulfuric acid action, was found to have less effect on the integrity than that of C-(N)-A-S-H gel, in which coarse gypsum crystalline grains formed and led to destructive stress in the hardened matrix.

In ref. [27], alkali-activated fly ash/slag cements were exposed to sulphuric acid to investigate anti-corrosion properties. The compressive strength change and mineralogical compositions, porosity and microstructure of individual layers of the corroded samples were tested. The results showed that the compressive strengths of all the samples decreased during acid attack. The most anti-corrosion mixture was the pure fly ash binder, which retained 83.5% strength after 28 days of exposure. Gypsum was only observed in the surface layer (0–5 mm) of samples. The samples with slag showed a notable pore structure change.

In the research studies referred to, where structural changes during degradation processes were monitored, the checking of strength decreases was rather marginal. However, in terms of possible practical use, in the case of concrete replacement with these materials, it is necessary to determine the degree of resistance at a level comparable to that of currently used materials. This research, therefore, focused on comparison in accordance with concrete testing standards, so that, if of interest, the results could be compared with different produced materials.

In this study, the aggressiveness of the environment was chosen according to the standard for concretes (EN 206-1). This standard specifies the marginal values of the concentration of sulfate ions for individual degrees of XA1-3 environmental impact. In an environment simulating acid rain, the concentration was chosen according to the pH of acid rain. The duration of exposure was chosen to be five months. This research is new, especially in considering the possibility of using CBPD and fly ash after denitrification using a selective non-catalytic reduction (SNCR) method as an admixture in alkali-activated GGBS-based materials, and in the selected concentration of sulfate ions, which was chosen according to EN 206-1. By using the standard for concretes, it is easier to assess the influence of the environment on the resistance of the prepared samples in terms of flexural and compressive strength. Using a combination of nitric acid and sulfuric acid simulated the acid rain effect. Graphical representation allowed comparison of the resistances of individual mixtures in different environments.

## 2. Materials and Methods

### 2.1. Materials

#### 2.1.1. Fly Ash (FA)

In the experiment, fly ash from the power plant in Ostrava-Třebovice, silica fly ash formed after denitrification by the selective non-catalytic reduction (SNCR) method, was used.

Denitrification is one of the secondary measures to reduce the nitrogen content, while in the flue gases, through a system of processes, nitrogen oxides are bound to the newly formed chemical compounds. These processes can be divided into selective catalytic reduction (SCR) and selective non-catalytic reduction (SNCR). In SNCR, nitrogenous compounds are concentrated in fly ash, which may contain ammonium salts, which may subsequently be released into the environment as ammonia during processing.

**Figure 1:**
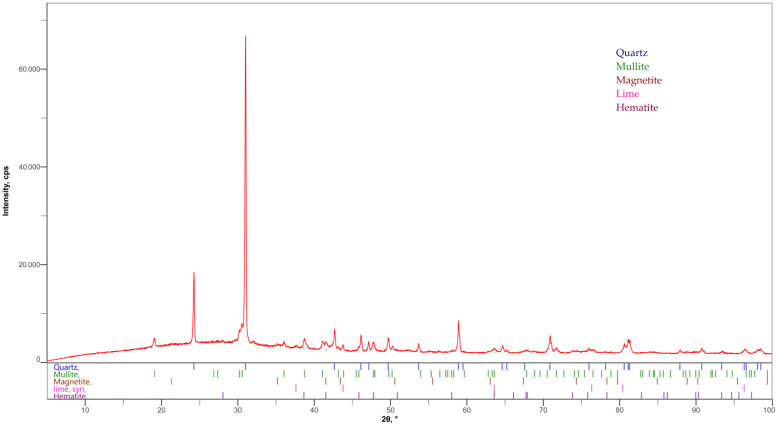
Mineralogical composition of FA determined by X-ray diffraction (XRD).

FA with the specific surface of 500 m^2^/kg was used. The specific weight of used fly ash was 2240 kg/m^3^. The content of ammonia released from the aqueous extract was 22.8 mg/kg. The chemical composition determined by X-ray fluorescence (XRF) is shown in Table 1. The mineralogical composition of fly ash was determined by X-ray diffraction. Figure 1 shows that SiO_2_ occurred in this fly ash mainly in the form of quartz {(PDF-01-070-7344)}. Other minerals present in this fly ash included mulite {(Al_6_Si_2_O_13_) (PDF-01-079-1458)}, magnetite {(Fe_3_O_4_) (PDF-01-071-6336)}, free lime {(PDF-01-082-1691)} and hematite {(Fe_2_O_3_) (PDF-01-085-0987)}. A typical amorphous arc as in ref. [28] was not clearly visible in these fly ashes, so it can be assumed that this fly ash had low reactivity, corresponding to the findings of [29], where the soluble amorphous phase content, determined by cooking in 4 M potassium hydroxide solution, was for FA only 4.17%. The total amorphous phase content calculated from the XRD analysis was 20% for FA.

#### 2.1.2. Ground Granulated Blast Furnace Slag (GGBS)

**Figure 2:**
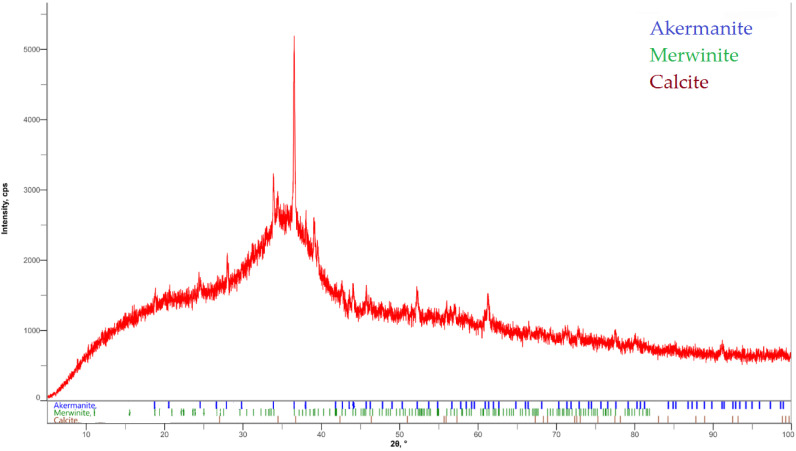
Mineralogical composition of BFS determined by X-ray diffraction (XRD).

For the experiment, finely ground granulated blast furnace slag was used. This slag has latent hydraulic activity and some pozzolanic characteristics from the reaction with portlandite [30] and has a specific surface of ≥400 m^2^/kg, with a specific weight of 2860 kg/m^3^. The percentages of individual oxides obtained by fluorescence spectrometer measurement are shown in Table 1. The mineralogical composition of GGBS in Figure 2 shows that mainly the minerals kermanite {(Ca_2_Mg(Si_2_O_7_)) (PDF-01-079-2424)}, merwinite {(Ca_3_Mg(SiO_4_)_2_) (PDF-01-074-0382)} and calcite {(CaCO_3_) (PDF-01-072-4582)} were present in this slag. The X-ray diffraction curve in Figure 2 shows that the slag was highly reactive because of a high proportion of amorphous phase content. The total amorphous phase content calculated from the XRD analysis was 80% for BFS [31].

#### 2.1.3. Cement By-Pass Dust (CBPD)

During the production of clinker, many dust particles are formed. Kiln dust is released into the air. If the appropriate technology is installed, dust particles from different stages of production are captured. It is a fine-grained material contained in the flue gas in a rotary kiln. It consists of a diverse mixture of calcined and non-calcined feed materials, fine clinker, fuel combustion by-products and condensed alkaline compounds. When installing a by-pass, the kiln gases are sucked out and, with rapid cooling, undesired gases condense on the surface of the dust particles. This material is a waste product. CBPD is only obtained under certain conditions if a bypass is installed. Installing a by-pass reduces unwanted impurities, such as chlorine and alkali. The quality of CBPD depends mainly on the combustion technology, the raw materials and the fuel used [32].

**Figure 3:**
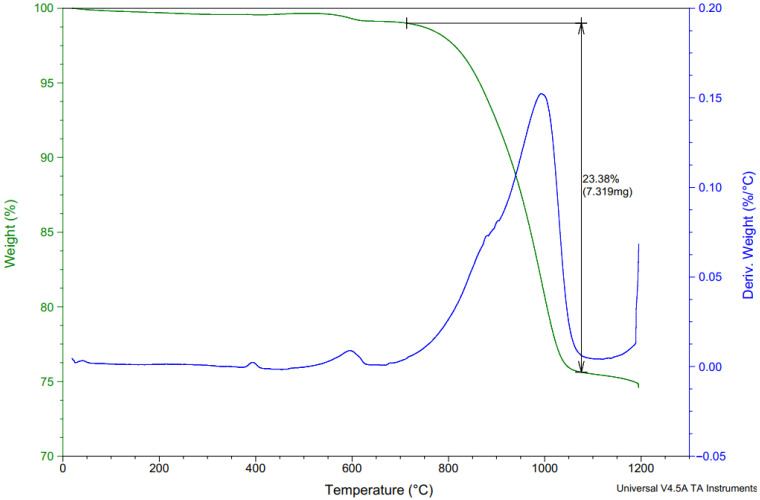
Thermal analysis of CBPD.

By-pass cement dust from the Horné Srnie cement plant was utilized in the experiment. The specific weight of the CBPD used was 2610 kg/m^3^. The percentages of individual oxides obtained by fluorescence spectrometer measurement are shown in Table 1. The chlorine content in CBPD was 10.49%. The phase composition of CBPD is given in Table 2. The LOI was 21.9% for CBPD. LOI is caused by the release of sylvite, which begins to be released at 700 °C and ends at 1100 °C. Another significant weight loss is caused by the decomposition of potassium sulfate, beginning around 1200 °C. LOI is also caused by the dehydroxylation of portlandite and the decomposition of limestone. These processes are shown in Figure 3. The chemical and phase composition of CBPD is taken from the report *“Deliverable D1.1 results from raw materials analyses”* within the GeoDust project. The evaluation of the measurements was carried out using the software HighScore, version 4.0 (Malvern, Worcestershire, UK).

CBPD can be used in alkali-activated materials mainly due to alkalinity, where it can partially replace the alkaline activator and thus significantly reduce the cost of composite production. However, the pH of the CBPD solution may not be basic enough to decompose the raw materials and subsequently form hydration products. Due to the presence of reactive lime in the mixture, a calcium silicate phase is formed [32].

#### 2.1.4. Activator—Anhydrous Disodium Metasilicate Na_2_SiO_3_ (A)

Anhydrous disodium metasilicate is a white crystalline material that contains a minimum of 44% SiO_2_. Anhydrous disodium metasilicate (Na_2_SiO_3_) produced by Penta Chemicals was used for the purposes of the experiment. This activator has a silicate modulus of 1. The basic chemical properties stated by the producer are given in Table 3, pH 12.5 was reached when dissolving 10 g/L at 20 °C; this value was stated by the producer [33].

#### 2.1.5. Standardized Sand

Standard sand CEN, EN 196-1 was used as filler in the experiment. It was natural quartz sand, which was formed of rounded particles and the silica content was min. 98%, 0/2 mm fraction and less than 0.2% moisture content [34].

### 2.2. Mixture

As a reference mixture, previously verified mixtures tested in previous studies [35,36,37,38] were used in the experimental component of the study. The basic mixture was derived from preliminary experiments, where the strength properties were monitored with different replacement quantities of GGBS with fly ash and CBPD, with the possibility of using different activators. Two activators were compared (Na_2_SiO_3_ and sodium water glass with a silicate modulus of 2). Better results were obtained with the Na_2_SiO_3_ activator; therefore, this activator was chosen for the next phase of the experiment. Within the reference mixtures, the activator dose was reduced by 15% for REF 2 and 30% for REF 3. For REC 1, the amount of activator was calculated from the total binder amount. For REC 2, the dose was reduced by 15% and for REC 3, the dose was reduced by 30% and was calculated only for GGBS. The reception of the raw material values is shown in Table 4. In these mixtures, a plasticizer was not used. The pH of the solution, prepared by dissolving anhydrous disodium metasilicate in water, ranged from 13.95 to 14.00 for all monitored mixtures.

### 2.3. Methods

#### 2.3.1. Preparation of Beams and Verification of Properties

To verify how the binder component of the alkali-activated-GGBS-based material would behave with admixtures of FA and CBPD, specimens measuring (40 × 40 × 160) mm were prepared. Preparation of mixtures followed standard requirements for the preparation of mortars and cements.

All necessary raw materials were weighed into the mixture with a precision of ±1 g. First, binder components, such as GGBS, FA and CBPD, were poured into the container, then activator and water were added. The mixing procedure was in accordance with the requirements of European standards EN 196-1 and EN 196-3. The binder component, together with the addition of activator and water, was mixed for 90 s at a low mixing speed according to EN 196-3. At the end of the cycle, mixing occurred according to the requirements of EN 196-1, with a total mixing time of 180 s, during which the sand was automatically dosed [34,39].

#### 2.3.2. Strength

To determine the basic strengths, samples were placed in a moisture box after filling the molds and subsequently demolded after 48 h and left until testing in a moisture box, with the exception of 1-day strengths, which were demolded after 24 h. To determine chemical resistance, the samples were placed in a humidity cabinet for 48 h after filling the molds, then demolded and placed in a water bath until placed in an aggressive environment. For each series of tests, 3 × samples measuring 40 × 40 × 160 mm were prepared. Strengths were determined using a Form+Test MEGA 100-300-10 DM1 hydraulic press with Proteus software. The strengths were determined according to the EN 196-1 standard. The determination of flexural strength was performed by uniform loading at speeds of (50 ± 10) N/s. Compressive strength was determined by uniform loading at speeds (2400 ± 200) N/s [34].

#### 2.3.3. Aggressive Environment

The resistance of the mixtures to aggressive environments was investigated in different environments that simulated different aggressive substances. According to the EN 206 [40] standard, solutions were prepared from concentrated sulfuric acid (H_2_SO_4_), so that the concentration of sulphate ions corresponded to the individual degrees of environmental influence XA1—XA3. From the stated range of sulphate ion concentration for a given degree of environmental influence, the highest content of sulphate ions in the solution was always chosen. For XA1 ion, the concentration was 600 mg/L, for XA2, 3000 mg/L, and for XA3, 6000 mg/L. Furthermore, solutions simulating acid rain were prepared, which were prepared by a combination of nitric acid (HNO_3_) and sulfuric acid (H_2_SO_4_) with a pH of 4.2. The samples were also placed in demineralized water, which simulated the effects of hungry waters. The pH of the prepared solutions was measured before storing the samples, according to which the concentration of sulfate ions was then replenished at regular intervals, so that the concentration of sulfate ions was at the desired value. The samples were stored in an aggressive environment after 28 days of maturation and remained stored in this environment for 5 months.

#### 2.3.4. Analytical Methods

Parts were cut from the selected specimens and thermal analysis and analysis of infrared spectroscopy with Fourier transformation were subsequently performed.

Thermal analyses were performed on an SDT Q600 from TA Instruments with a heating rate of 10 °C/min to 1000 °C with a 2 min hold at this temperature. The evaluation was performed using the TA Instruments software, Universal Analysis 2000.

Infrared spectroscopy was performed on a Nicolet iS50 FT-IR instrument. The experiment used a sample compartment iS 50 ATR, a detector DTGS ATR, and a beam splitter kBr. The measurement took place in the range of 400–4000 cm^−1^.

X-ray diffraction was performed on a Rigaku MinyFlex instrument in the range of 5–100° 2 theta at a speed of 3 °/min. A D/tex Ultra 2 detector was used in the measurement and the 15 mA lamp voltage was 40 kV. The calculation of the amorphous phase in the input raw materials was performed using PDXL2 software (Rigaku).

Sample surfaces were imaged using a JEOL JSM-7610F Plus Scanning Electron Microscope (SEM) (JEOL, Akishima, Japan) in secondary (SE) and backscattered electron (BSE) mode. The chemical composition of MAO was studied using an SEM-attached dispersive X-ray spectrometer (EDS, ULTIM MAX 65 mm 2, Oxford Instruments, UK). Sputtering was performed with Quorum 150V ES (Quorum, UK), 20 nm Pt.

Mixtures labeled REC 2 and REF 2, after 5 months of storage in an aggressive environment, were used for the analytical methods. 5 mm plates were cut within these samples. In the case of samples stored in XA3 solution, a severely degraded surface layer was removed from the cut sheet, which was subsequently tested separately. These plates were crushed and ground to a fine powder and subsequently tested by thermal analysis and infrared spectroscopy with Fourier transform.

The Fourier transform is a mathematical tool. When using this tool, any function can be decomposed into the sum of sinusoidal basis functions. Each of these basic functions is a complex exponential of a different frequency. The Fourier transform provides a unique way to look at any function—as the sum of simple sinusoids.

The Fourier transformation equation is defined according to Equation (1) [41].

(1) $$F\{g(t)\}=G(f)=\int_{-\infty }^{\infty }g(t)e^{-2\pi ift}dt$$

## 3. Results and Discussion

### 3.1. Determination of Basic Physical-Mechanical Properties

**Figure 4:**
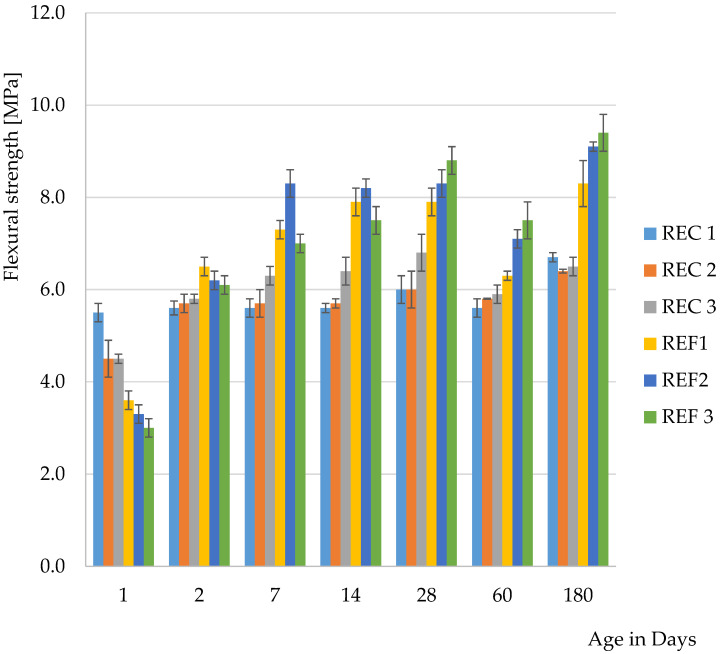
Flexural strength of mixtures.

**Figure 5:**
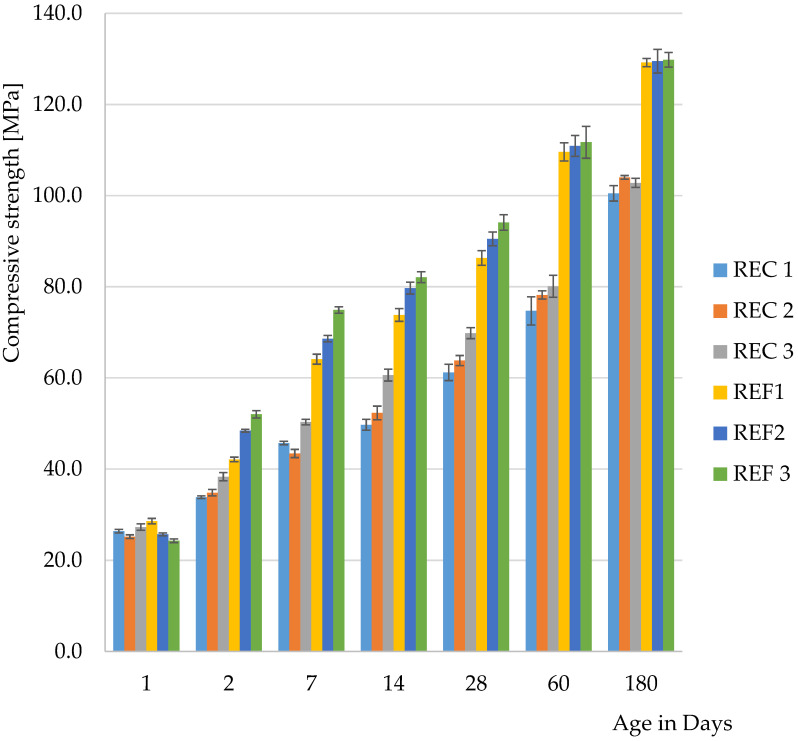
Compressive strength of mixtures.

The determination of basic physical—mechanical properties are shown in Figure 4 and Figure 5.

From the results of the basic physical-mechanical properties so far, it was possible to observe the acceleration of hydration processes in mixtures where cement by-pass dust had been used. This manifested itself mainly in the one-day flexural strength. The faster hydration process was attributed to CBPD, because, when the use of fly ash after denitrification was verified, no acceleration of solidification was observed.

From the second day of maturation, higher compressive strengths were achieved for all reference mixtures than for mixtures with admixtures. After 180 days of maturation, the compressive strengths of the reference mixtures were almost identical and were around 130 MPa. For mixtures with admixtures, these strengths were around 100 MPa.

In terms of the amount of activator used, the lowest dose of activator appeared to be the optimal amount. The only exceptions were the one-day strengths, where higher values were achieved for mixtures with the highest activator content, which was especially evident in the compressive strengths. This development was expected, because the higher alkali content caused a higher pH in the mixtures and the reactions proceeded faster. The flexural strengths after 28 days reached the highest values in the reference series with the lowest activator dose. The highest strength was also achieved for the mixtures with the lowest activator dose after 28 days.

The authors in ref. [42] studied the effect of CBPD on alkali-activated blast furnace granulated slag-based materials. Two mixtures with different amounts of sodium oxide (4 and 6)% were monitored. The strength with 6% Na_2_O reached about 57 MPa after 28 days and the mixture with 4% Na_2_O reached a strength of about 43 MPa. Flexural strengths were in the range of 6–7 MPa after 28 days of maturation [42].

In ref. [32], the effect of slag replacement by CBPD was monitored in the range 5 to 25%. Sodium carbonate and sodium water glass were used as activators in the mixture. When CBPD is mixed with water, calcium hydroxide is formed in the mixture, which then reacts with sodium carbonate to form sodium hydroxide and limestone according to Equation (2).

Ca(OH)_2_ + Na_2_CO_3_ → 2NaOH + CaCO_3_, (2)

By adding up to 20% CBPD, the strength characteristics were improved; with 25% replacement, these parameters had already deteriorated, which was mainly related to the short workability time and the associated homogenity of the sample. Flexural strength was observed to decrease after 28 days of maturation in 100% GGBS. The authors attributed this decrease to the chemical shrinkage of this mixture. CBPD also had a positive effect on shrinkage, when CBPD swelled due to dead-burnt lime which is contained in CBPD. It was found that full expansion occurred within three days. On the other hand, the 100% GGBS mixture shrank despite being stored in water, which was due to the high atomic density of the resulting hydration product. As a result, water cannot penetrate the structure during curing and self-drying occurs. This process is called autogenous shrinkage and occurs throughout the system [32].

In ref. [43], the influence of fly ash on the physical-mechanical properties of alkali activated materials based on GGBS was studied. The reference series was identical in terms of bulk materials, only a smaller amount of mixing water was used. In the current research, this mixture is referred to as REF 1. When comparing 28-day strengths, slightly higher values were achieved in the current research. In the case of flexural strength, better results were obtained in the previous research, the strength achieved being 8.9 MPa. Compressive strengths were higher for mixtures with 20 and 30% GGBS replacement with fly ash; this applied both to fly ash before denitrification and after denitrification by the SNCR method, which was also used in the current research [44].

The authors of [43] studied the effect of silicate modulus and Na_2_O content on the properties of alkali-activated GGBS. The Na_2_O content in the mixtures was 2–6% and the silicate modulus was 0.6, 0.9 and 1.2. The maximum compressive strength after 28 days of maturation was achieved for a mixture with 3% Na_2_O and with a silicate modulus of 0.6. After 180 days of maturation, the highest flexural strength was achieved for a mixture with 2% Na_2_O with a silicate modulus of 0.6. It was found that after 7 days of maturation, higher strengths were achieved by mixtures with a higher proportion of Na_2_O, but after 28 days, the situation reversed and there was found to be a decrease in strength. In contrast to flexural strengths, the compressive strengths increased throughout the maturation period and the highest values were always achieved with the highest Na_2_O content and the highest silicate modulus. The lowest strengths were achieved with mixtures with the lowest Na_2_O content and the smallest silicate modulus [43].

In ref. [32], the authors stated that there was an improvement in the strength characteristics after 7 and 28 days of maturation, but in this research, there was an improvement only in 1-day flexural strength and for pressure there were only minimal differences between mixtures. This may be due to an increase in alkalinity in the first hours of maturation due to the use of CBPD, but then the content of additives has a negative effect on the strength characteristics. When the effect of fly ash, including fly ash after denitrification by SNCR, was monitored in ref. [34], higher compressive strengths were achieved after 28 days by mixtures with a partial replacement of GGBS with fly ash up to 30% by weight than in the reference series (100% GGBS).

Achieving higher strengths in ref. [32] was associated with a significant increase in pH, with the pH of sodium carbonate hovering around pH 11.13, while the combination of sodium carbonate with 5% CBPD increased the pH of the solution to approximately 13, and with increasing CBPD replacement, the pH increased further.

In this research, the pH of the alkaline solution in the mixture was around 13.95, which is sufficient for the dissolution of the components and the forced formation of hydration products. Therefore, the effect of CBPD was manifested only in 1-day strengths, during the first hours of maturation, when the dissolution of the components was even more intense.

### 3.2. Results of Aggressive Environment

**Figure 6:**
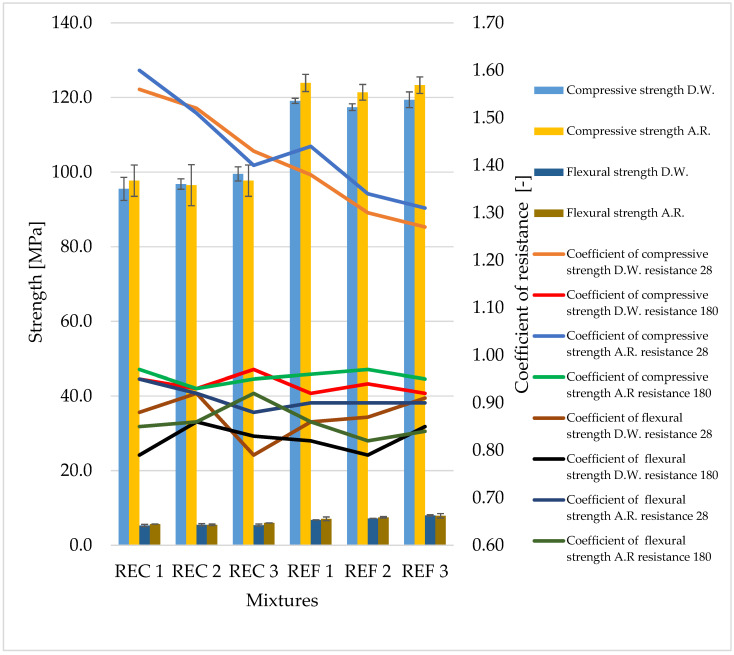
Strength characteristics of prepared mixtures in aggressive environments A.R and D.W.

**Figure 7:**
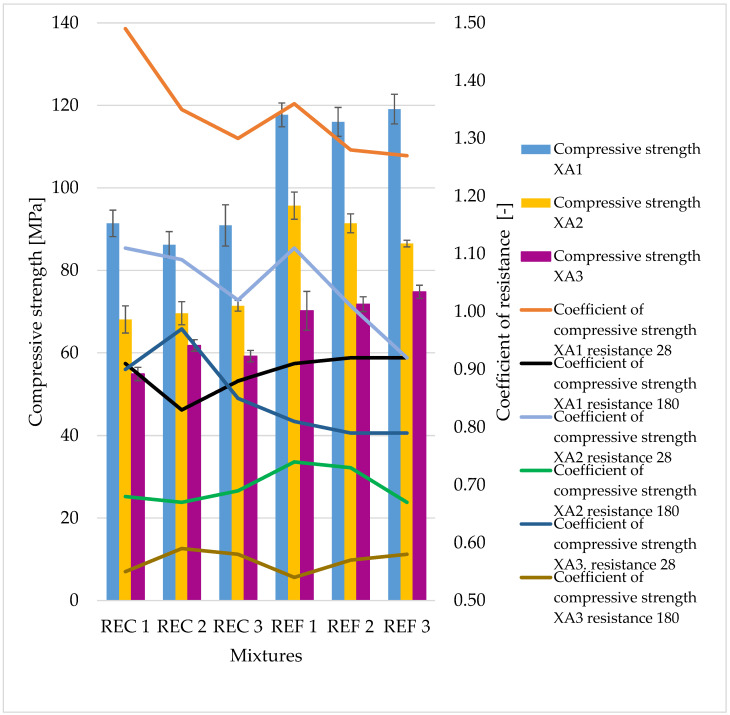
Compressive strengths of prepared mixtures in aggressive environment XA1-3.

**Figure 8:**
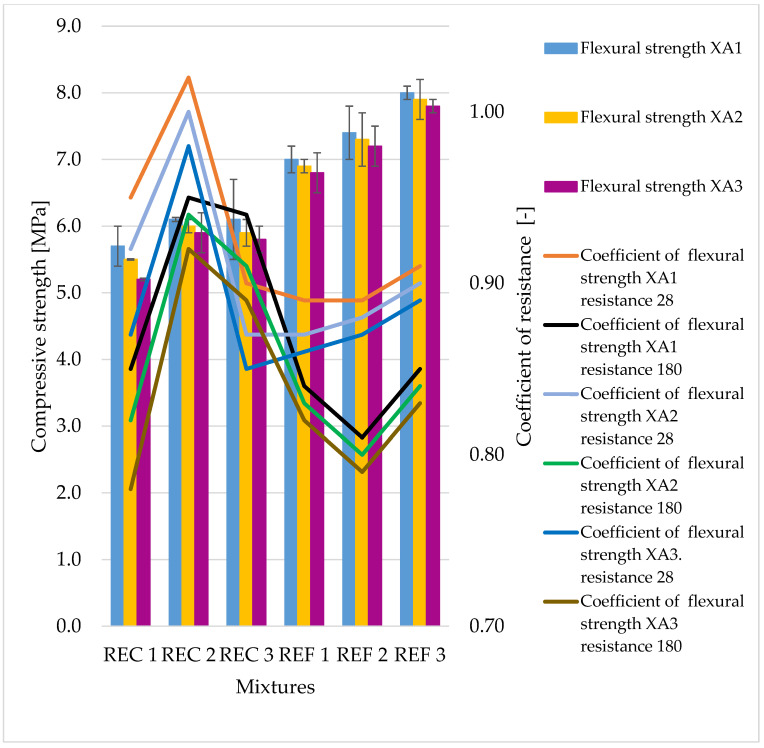
Flexural strengths of prepared mixtures in aggressive environment XA1-3.

**Figure 9:**
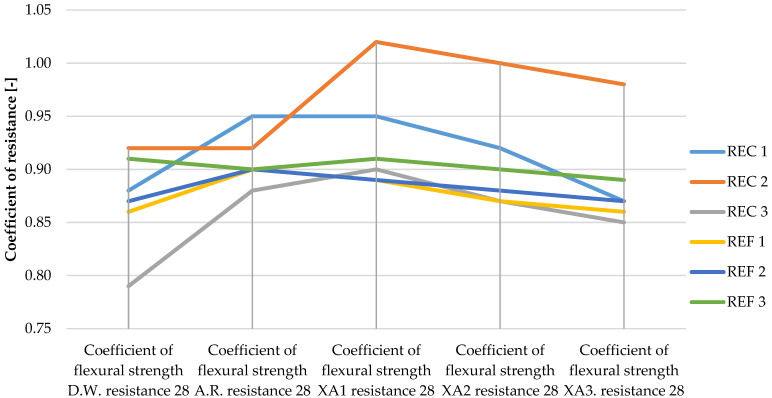
Coefficients of flexural strength in relation to 28-day strengths of samples in aggressive environments.

**Figure 10:**
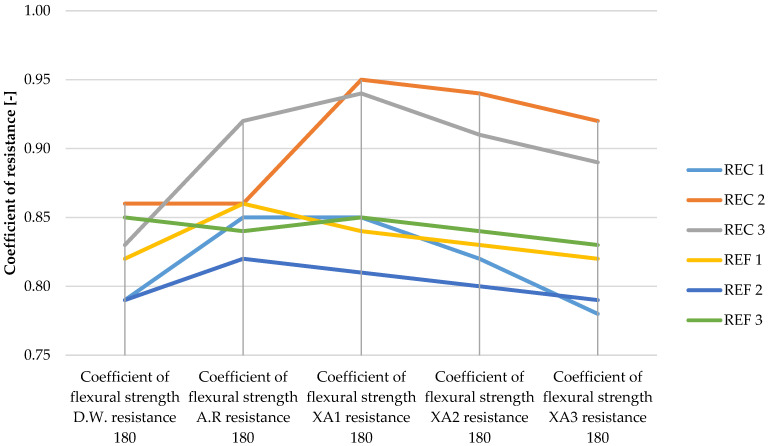
Coefficients of flexural strength in relation to 180-day strengths of samples in aggressive environments.

**Figure 11:**
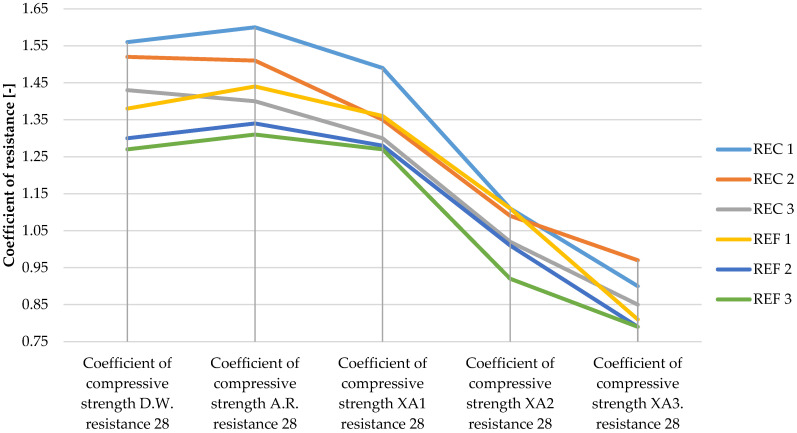
Coefficients of compressive strength in relation to 28-day strengths of samples in aggressive environments í.

**Figure 12:**
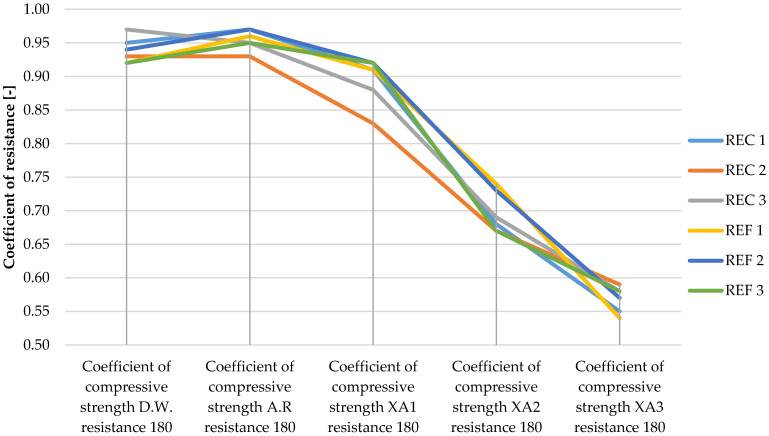
Coefficients of compressive strength in relation to 180-day strengths of samples in aggressive environments.

**Figure 13:**
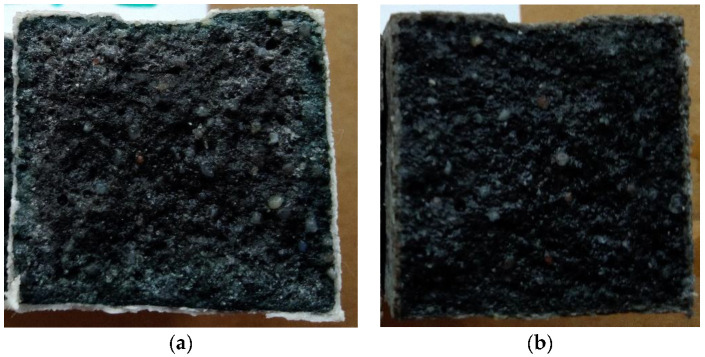
Surface degradation in mixtures (**a**) REF 2, (**b**) REC 2 deposited in XA2 solution.

**Figure 14:**
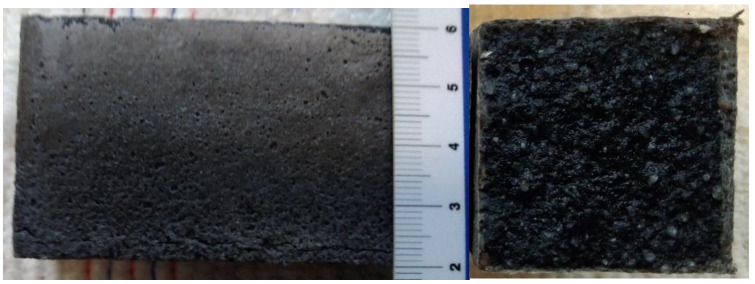
Surface degradation of mixture REC 2 deposited in XA3 solution.

**Figure 15:**
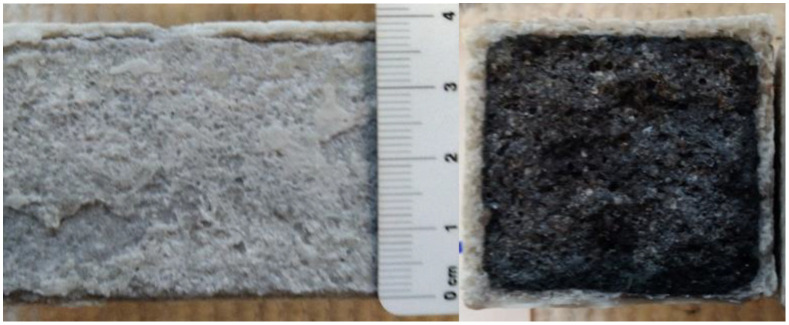
Surface degradation of mixture REF 2 deposited in XA3 solution.

The determined basic physical and mechanical properties of the mixtures, which were stored in an aggressive environment for 150 days, are shown in Figure 6, Figure 7 and Figure 8. The resistance coefficients were also calculated. These coefficients were calculated for 28 days of strength, as well as for the same age reference series, which was stored in a water bath for 180 days. The resistance coefficient was calculated as the ratio between the strength value of the sample stored in the aggressive environment and the reference series and was related to the 28-day or 180-day reference strengths. The resistance coefficients are shown separately in Figure 9, Figure 10, Figure 11 and Figure 12. Figure 13, Figure 14 and Figure 15 show mixtures of REC 2 and REF 2 after 5 months of storage in an aggressive environment. Table 5 explains the markings used.

**Table 5:** Explanation of used markings of the aggressive environment.

Mark	Description	pH	Note
D.W.	Demineralized water	6.81	
A.R.	Acid rain	4.25	Prepared from nitric acid and sulfuré
XA1	Degree of environmental influence according to EN 206-1	1.99	Concentration of sulfate ions 600 mg/L
XA2	Degree of environmental influence according to EN 206-1	1.55	Concentration of sulfate ions 3000 mg/L
XA3	Degree of environmental influence according to EN 206-1	1.27	Concentration of sulfate ions 6000 mg/L

Demineralized water has a minimal effect on compressive strength; the coefficient after 180 days ranged from 0.9 to 1.0. The REC 3 mixture reached the best value. In comparison with the strength after 28 days of maturation, an increase in strength can be seen even in this environment (the coefficients ranged from 1.2 to 1.6). Mixtures with admixtures performed best in this environment.

In the case of flexural strengths, the effect of demineralized water was greater; the coefficient after 180 days was at the level of 0.75–0.85, so the strengths decreased by up to 25%. The decrease in strength was also evident in comparison with the values after 28 days of maturation; this was around 10%—the most striking decrease was 20% (mixture REC 3).

In the environment simulating acid rain, the values of the compressive strength coefficients were at a similar level to demineralized water. In the same way, the results and the values of flexural strengthwere similar, but the highest value of the coefficient of all tested mixtures was reached by mixture REC 3.

The anticipated effect of the amount of activator on decrease in strength was not observed.

Previous research [30], using the same raw materials (GGBS and fly ash) which were activated by sodium water glass with a silicate modulus of 2, achieved a decrease in flexural strength of the bend of 20% after 28 days of storage in demineralised water for 10% FA, 20% FA and 20% FAD. The coefficients under pressure were around 1.0.

Previous research [45], using the same raw materials (GGBS and fly ash), which were activated with potassium water glass with silicate modulus of 2, showed almost no effect of demineralized water on the compressive strength. On the other hand, there was a significant effect of demineralized water on the flexural strength. The biggest decrease was achieved by the GGBS mixture, which can be explained by the fact that this mixture had the highest amount of calcium, which was the most prone to leaching. However, if we accept the same condition as for resistance to freeze-thaw, that the drop in strength must not fall below 75% of the reference series, then all the mixtures met this condition [45].

In ref. [44], the influence of fly ash on the physical-mechanical and durability properties of the alkali-activated materials based on GGBS was studied. The reference series was identical in terms of bulk materials, only there was less water; in the current research study this mixture is called REF 1. Resistance to aggressive environments simulated by demineralized water showed a negative effect of fly ash on the resulting values. The highest coefficient of resistance to aggressive environments was found for the mixture prepared only from blast furnace granulated slag without the addition of fly ash. The lowest flexural strength coefficient of resistance to aggressive environments was achieved by the mixture with 10% slag replacement by fly ash after denitrification. Although a different effect of fly ash on the resulting properties was found, no 25% decrease in strength was found in any of the mixtures, and all mixtures therefore met the resistance conditions [44].

The authors in ref. [46] studied the properties of alkali-activated materials with different ratios of slag and fly ash. Compressive strengths in three different environments were monitored depending on the age of the samples. Individual samples at different ages (7, 28 and 60) were placed in demineralized water, immersed to 1 mm in demineralized water and left in air at 22 ± 2 °C, and subsequently tested after 7, 28 and 60 days. Regardless of the ratio between slag and fly ash, age or exposure time, the lowest strengths were always achieved for samples immersed in 1 mm of demineralized water. In the mixture where there was 100% by weight of slag, there were only minimal differences between the individual deposits after 28 days of maturation and subsequent exposure. In terms of results, the strength increases at the time of 60 days when immersed in demineralized water are interesting, while the strengths decreased when stored in demineralized water for 28 days. This applied to mixtures with 100, 75 and 50% by weight of slag. In the mixture with 25% by weight of slag, all the strengths of samples stored in demineralized water increased, regardless of the maturation time or the duration of exposure [46].

In terms of resistance to hungry water, simulated by the action of demineralized water, it would be interesting to monitor resistance in the longer term, because in ref. [46] there were increases in strength at a storage time of 60 days compared to a storage period of 28 days. However, the question is whether these increases would continue even after prolonged exposure to demineralized water, or whether there would be a significant decrease in strength, caused by decalcification of the hydration product C-(A)-S-H gel.

Significant decreases in compressive strengths were observed with increasing aggressiveness of the environment. From the trends in individual coefficients, it can be concluded that in the XA1 environment the samples lost about 10% in compressive strength, the worst of which was REC 2 where the decrease was close to 20%. The XA2 environment caused a decrease in compressive strength of 25–35%. According to the assumptions, the strenghts of samples were the worst in the XA3 environment, where the decreases in strengths were about 40–45%.

In terms of the assessment of mixtures with and without admixtures, we can say that the admixtures had essentially no effect on the strength parameters when stored in the given environments; although they achieved lower strengths, the percentage strength losses were approximately the same.

The same dependence of flexural strength as of compressive strength of the samples was observed, with the strengths of all mixtures decreasing with increasing aggressiveness of the medium. The maximum decreases in strengths were around 20%, which was significantly less than that of the compressive strengths, where the maximum drops were around 45%. From the curves of the resistance coefficient, it is possible to infer a certain uniformity of results within individual mixtures, with quite uniform decreases depending on the aggressiveness of the environment. For admixtures, the different resulting values of the coefficients of REC 2 are very interesting. Surprising too are the relatively low values of the flexural strengths of all reference mixtures and especially the significant decrease in REF 2 with a medium dose of activator.

The trends in the resistance coefficients for flexural strength did not show significant decreases caused by different aggressiveness of individual environments. However, significant declines in compressive strength coefficients of resistance in relation to degrees of environmental influence XA2 and XA3 were clearly visible. The coefficients of compressive strength also showed an increase in the coefficients relative to the 28-day reference series, suggesting that hydration reactions were taking place even in a milder aggressive environment, which were able to offset the decreases in strengths in the degradation layers. This phenomenon was notable in the D.W., A.R. and XA1 conditions.

Similar results were obtained in ref. [16], where increases in strengths in aggressive environments were also recorded.

The authors of [47] also studied the resistance of alkali-activated materials to sulfuric acid. A sulfuric acid solution of pH 1 was used and samples were stored in this solution for 70 days. GGBS was in a minor proportion in the mixture, therefore the mixtures had to be warmed for 24 h. Mixtures based on microsilica and rice husk ash were heated up to 80 °C and mixtures with slag admixture were heated to 60 °C at 80% relative humidity. The highest compressive strength, 58 MPa, was achieved with a mixture with microsilica. While the best resistance (78%) to sulfuric acid was achieved with a mixture of rice husk ash. The difference in the coefficient of resistance was about 10% smaller for the microsilica-based mixture than for the slag-based mixture. For the mixture based on rice husk ash, strengths were 20% smaller than for the slag-based mixture [47].

The authors in ref. [48] monitored the resistance of alkali-activated fly ash-based pastes with partial (%) replacement of GGBS by the fly ash in a 10% sulfuric acid solution. Samples were stored in solution for 28 and 56 days. The highest strength was achieved for the 50% mixture (50% of GGBS was replaced by the fly ash). After 56 days of maturation, the highest compressive strength was achieved for the 30% mixture; it recorded the highest increase in strength in the range of 28–56 days. The largest decreases in strength were recorded for the 70% mixture, both after 28 days and after 56 days. In terms of the residual strength ratio, the best resistance was achieved with the 90% mixture. The paste with 50% had higher residual strengths than the paste made of traditional Portland cement. The 10% and 50% mixtures achieved higher residual strengths than 70% mixtures and traditional Portland cement. This was due to the fact that a 50% mixture had a denser matrix and a small amount of permeable cavities, whereas, in the 90% mixture this was due to the high resistance of the hydration product of the N-A-S-H gel to the action of acids, even though the mixture contained a high proportion of permeable cavities. The 70% mixture had more voids than the 50% mixture and the amount of N-A-S-H gel was smaller, while the amount of N-C-A-S-H gel, which had less acid resistance, was greater than for the 90% mixture [48].

The authors in ref. [27] also studied the resistance to a 3% sulfuric acid solution of alkali-activated pastes with different proportions between GGBS (S) and fly ash (F). The highest strength was achieved for the S70/F30 mixture. This mixture also resulted in the highest decrease in strength after 28 days of storage in a 3% sulfuric acid solution. The residual strength of the mixture was 53.1%. For the mixtures S100 and F70/S30, the residual strengths were around 75% of the reference series. The highest resistance was achieved with the F100 mixture, where the residual strengths were around 84% of the reference series [27].

The authors in ref. [49] studied the resistance to various types of 5% acid solutions (hydrochloric, sulfuric and nitric) of alkali-activated concretes based on GGBS. Two concentrations of alkaline solutions were monitored. The results showed that mixtures with a higher concentration of alkaline solution achieved a higher resistance to sulfuric acid after 56 days. The resistance to hydrochloric acid after 56 days was also greater for mixtures with a higher concentration of alkaline solution. The largest decrease in all the monitored samples was achieved for the mixture with the lowest binder content per 1m^3^ and with the lowest concentration of alkaline solution [50].

The authors in ref. [16] monitored the resistance of alkali-activated mortars to acid solutions with a pH value of 3. Samples of Portland cement were also tested. Resistance parameters against the three types of acids (hydrochloric, nitric and sulfuric) were monitored. In the case of cement mortars, there were only slight changes in the compressive strength of the samples exposed to the acids. For alkali-activated mortars, there were slight decreases in all samples stored in water or acid solution after 30 days of storage. After 150 days of storage in acidic solutions, all strengths were higher than the original series, which, according to the authors, was due to the fact that hydration processes still took place in a healthy core and these were able to compensate for the reduction in strength in the degraded layer on the surface. The highest increase in strength after 150 days was recorded for samples stored in water [16].

Figure 13, Figure 14 and Figure 15 show the degradation of the surface due to the action of sulfate ions, which resulted in the formation of calcium sulphate. Calcium sulphate formation occurred due to the descaling of hydration products based on the C-(A)-S-H gel.

The peeling of the surface degraded layer is clearly visible in Figure 15 and was caused by the formation of calcium sulphate, which increased its volume by up to 125% [27].

Figure 15 is an optical microscope image showing calcium sulphate crystals from a peeled degraded layer of REF 2 after 5 months of storage in XA3.

**Figure 16:**
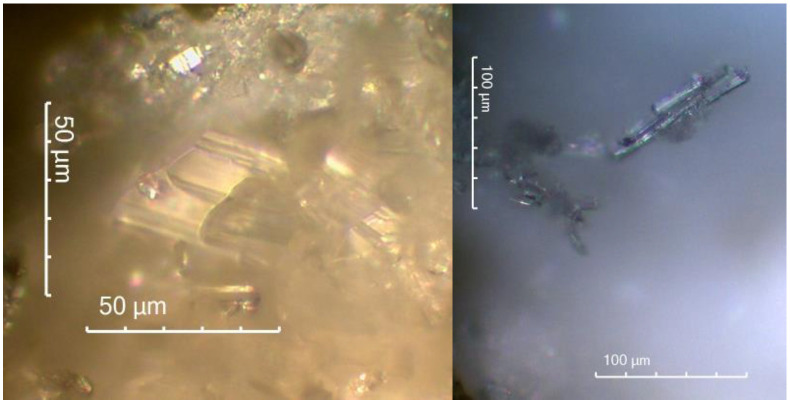
Calcium sulphate crystals on the surface of degraded layer REF 2 mixture deposited in XA3.

The authors in ref. [51] also identified calcium sulphate as a degradation product, monitoring the resistance of alkali-activated pastes to magnesium sulphate, and showed similar calcium sulphate crystals from scanning electron microscopy as in Figure 16 [29].

### 3.3. Analytic Methods

**Figure 17:**
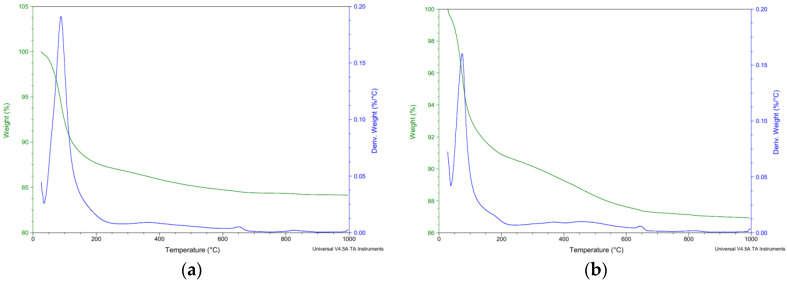
Thermal analysis reference series (**a**) REF 2 (**b**) REC 2.

**Figure 18:**
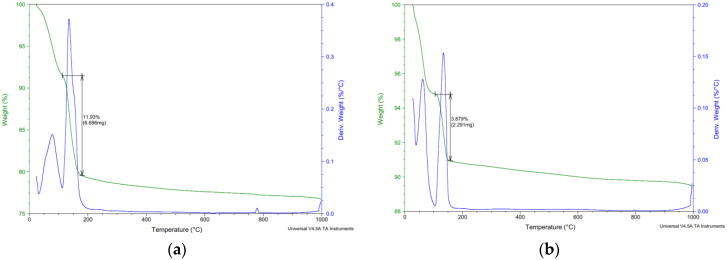
Thermal analysis—XA3—(**a**) REF 2 (**b**) REC 2.

**Figure 19:**
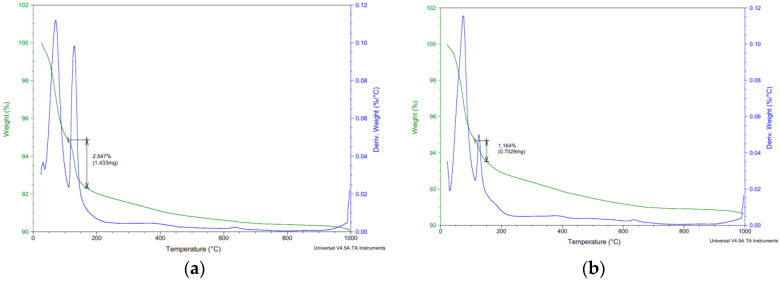
Thermal analysis—XA3—(**a**) REF 2 (**b**) REC 2.

**Figure 20:**
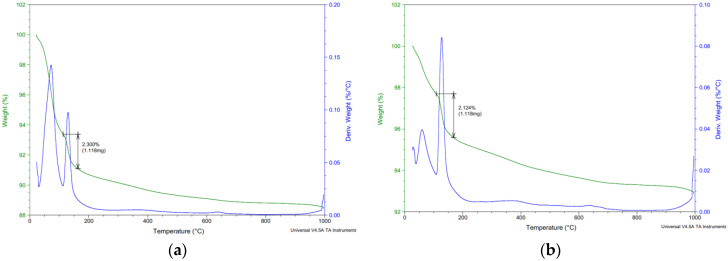
Thermal analysis—XA2—(**a**) REF 2 (**b**) REC 2.

As part of the analytical methods, the surface layers were tested in the depth area up to 5 mm. All surface layers in all aggressive deposits were tested. Figure 17, Figure 18, Figure 19 and Figure 20 show selected analyses of the thermal processes of the surface layers. As no degradation products or significant deviations from the reference series were found for several series, they are not listed here.

#### 3.3.1. Thermal Analysis

Thermal analyses were performed on all samples, but the results show only analyses that differed from the reference series.

Figure 17 shows the thermal analysis curves of the reference series. The weight loss curve shows a release of low-temperature water in the region of 0–200 °C with slow releas of water in the region of 200–600 °C at higher temperatures [50]. Decomposition of limestone was evident in the range of 600–650 °C.

Figure 18, Figure 19 and Figure 20 show the results of thermal analysis of samples stored in the aggressive environments XA3 and XA2. Figure 19 shows the analysis of the peeled degraded layer of samples stored in XA3. These curves show the release of free water up to 100 °C. In the range of 100–180 °C, the decomposition of the calcium sulfate dihydrate degradation product was clearly visible, where the calcium sulfate dihydrate was converted to calcium sulfate hemihydrate by partial dehydration. The curves do not show the conversion of calcium sulphate hemihydrate to anhydrite.

Figure 19 and Figure 20 also show the release of free water up to 100 °C. In the range of 100–180 °C, there was decomposition of calcium sulphate dihydrate to calcium sulphate hemihydrate.

The total amount of calcium sulphate contained in the degraded layer was 76.01% for REF 2 and 16.23% for REC 2. This large difference can be seen in the visual evaluation of Figure 14 and Figure 15 where the manifestation of calcium sulfate is much more pronounced in the REF 2 in Figure 14. When storing samples in XA2, the amount of calcium sulfate in both mixtures was approximately the same, 14.65% in REF 2 and 13.53% in REC 2.

In the area of 400 °C, a small weight loss was evident, which the authors in ref. [29] identified as resulting from decomposition of sodium carbonate.

The authors in ref. [29] studied the resistance of alkali-activated slag-fly ash pastes to the action of sodium sulfate and magnesium sulfate. No degradation product was detected under sodium sulphate treatment, while calcium sulphate was detected as a degradation product under magnesium sulphate, which was identified by XRD, FTIR and thermal analysis. Decomposition of calcium sulphate dihydrate occurred at 110–150 °C when calcium sulphate hemihydrate was formed by partial dehydration. Subsequently complete dehydration to anhydrite occurred in the range of 150–200 °C; this conversion was difficult to detect due to overlap with other phases contained in the paste [29].

#### 3.3.2. FTIR Analysis

Samples of the second series were selected for FTIR analysis. Samples stored in XA1-3 medium were monitored and compared with a reference mixture stored in a water bath.

From the analysis of samples designated as XA3 DEG, XA3 and XA2, calcium sulphate dihydrate could be identified as a degradation product after 150 days of storage in an aggressive environment. The presence of calcium sulphate dihydrate was confirmed by tension OH bonds in the region of 3515–3396 cm^−1^ and bending bands in the region of 1682 and 1619 cm^−1^. Especially in the case of the XA3 DEG sample, the SO_4_ stretching bonds in the areas of 1100, 598 and 667 cm^−1^ were clearly visible [29].

The authors in ref. [29] also performed an XRD analysis and identified the degradation product as calcium sulfate dihydrate.

Samples stored in XA1 had a normal waveform and were similar to the reference series.

In the analysis of samples XA2 and XA3, the shape of the main peak in the range 950–1100 cm^−1^ indicated the possible coexistence of the main hydration product of the C-(A)-S-H gel and the calcium sulfate dihydrate product of degradation.

In the area of 3380–3395 and 1640 cm^−1^, water stress (O-H) and deformation (H-OH) bands occurred. These areas were caused by the presence of chemically bound water in the hydrated alkali-activated materials in different spectra. Absorption bands around 1478 cm^−1^ corresponded to O-C-O vibrations in carbonates. The main absorption band of the reaction products was located at around 954 cm^−1^ in all mixes, which were assigned to the asymmetric stretching vibration of Si-O-T (T = tetrahedral Si or Al) terminal (non-bridging) bonds, indicating that the main reaction product was a chain structured C-(A)-S-H type gel. The area moving around 418–440 cm^−1^ represented Si-O-Si and O-Si-O bonds [51,52,53,54,55].

**Figure 21:**
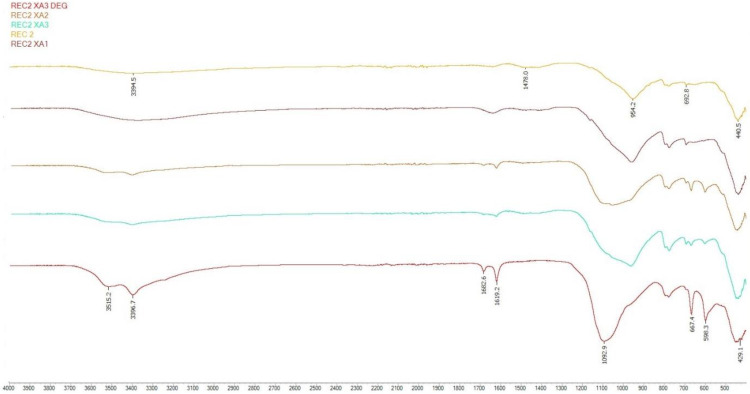
FTIR analysis of REC 2.

**Figure 22:**
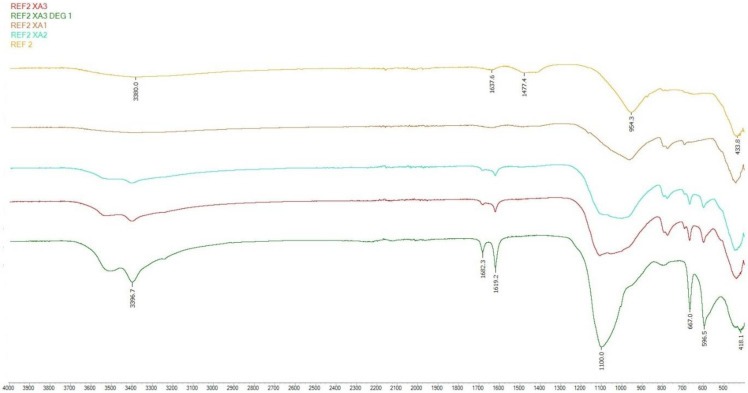
FTIR analysis of REF 2.

Figure 21 and Figure 22 clearly show a change in the main peak, which represents the asymmetric stretching vibration of Si-OT (T = tetrahedral Si or Al) in the reference series to peak SO_4_ stretching bonds, which represent the degradation product calcium sulphate dihydrate; this transformation was most pronounced for the strongly degraded layer, which was clearly visible, especially as shown in Figure 14. In other analyzed samples from the shape of the main peak the possible coexistence of Si-O-T and SO_4_ bonds can be inferred.

#### 3.3.3. SEM Analysis

**Figure 23:**
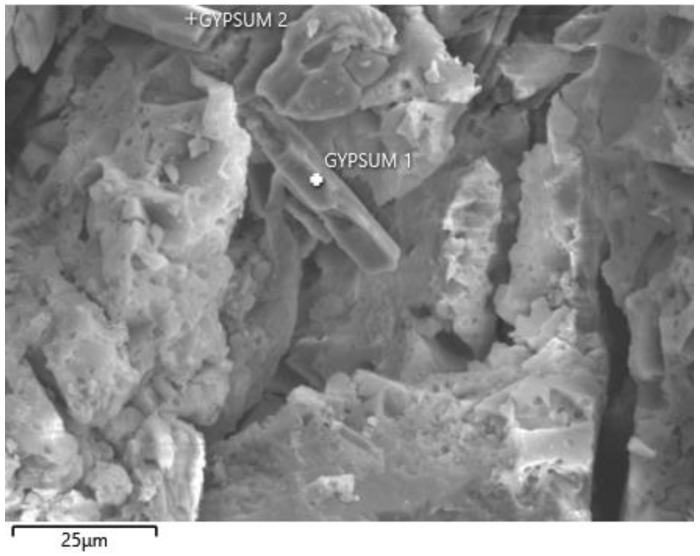
SEM analysis of REC 2.

**Figure 24:**
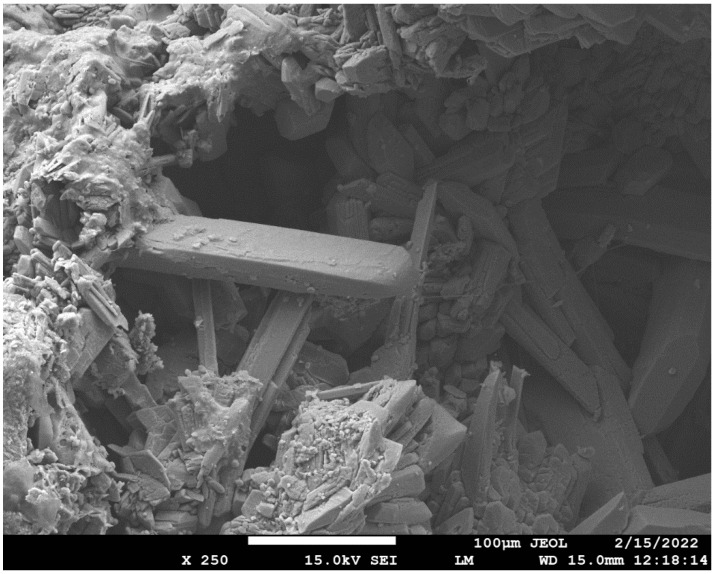
SEM analysis of REF 2.

**Figure 25:**
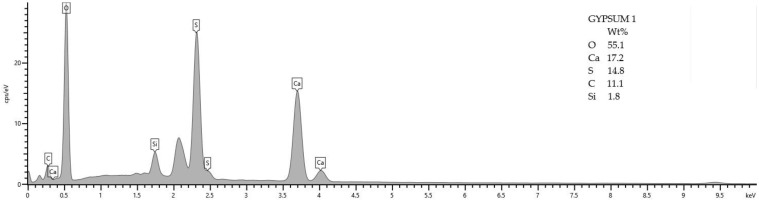
Chemical analysis of the gypsum crystal 1 of Figure 23.

SEM analysis was performed on samples after 5 months of storage in XA3 medium for mixtures REC 2 and REF 2. The analysis was focused mainly on the identification of degradation products. The results of the SEM analysis are shown in Figure 23 and Figure 24. The chemical composition of the gypsum crystal is shown in Figure 25.

Figure 23 and Figure 24 show the results of the SEM analysis. Gypsum crystals are clearly visible in both pictures. In Figure 24 we see a larger amount of gypsum crystals, which was confirmed by thermal analysis of these samples, when the mixture REF 2 contained a larger amount of gypsum than the mixture REC 2. In the evaluation, a chemical analysis of gypsum crystal (Gypsum1) was performed, as shown in Figure 23. This analysis is shown in Figure 25.

## 4. Conclusions

Admixtures in the long term worsened the strength parameters, and reduced the final strength. Even so, the compressive strengths of the mixtures with admixtures were around 100 MPa.

The largest decreases in strengths when stored in an aggressive environment were observed for compressive strengths in the XA3 solution; this was to be expected because this environment contained the highest concentration of sulfate ions. These decreases amounted to up to 45% of the same old reference series.

When comparing mixtures with and without admixtures, it was not entirely clear which mixtures showed better resistance to aggressive environments, because the differences in resistance coefficients relative to 180-day strengths were small and were different for individual mixtures.

Within the analytical methods, calcium sulphate dihydrate was identified as a degradation product, especially in the XA3 environment. This was determined by FTIR and thermal analysis.

Using thermal analysis, a significantly higher amount of gypsum was found in the strongly degraded layer in the REF 2 mixture, when the gypsum content of this mixture was approximately 60% higher than in the REC 2 mixture. However, this significant difference was not reflected in the compressive strengths of the same old reference series.

The compressive strength results indicate that the reactions in the healthy core continued even when stored in a highly aggressive environment, which was evident in the resistance coefficient relative to the 28-day reference series. In addition to the XA3 environment, this coefficient was above 1, which indicates that reactions took place even after being placed in an aggressive environment.

In the next phases, the experiments will focus on monitoring the changes in the structure during the degradation process and on the depth of degradation through the element. The results obtained so far show that the tested mixtures are resistant to the effects of hungry waters and acid rain and could therefore be applied in these environments. According to the current results, the REC 2 and REC 3 mixtures are resistant to the action of the aggressive environments XA1-XA3 for flexural strength, but significant decreases in compressive strengths were found; therefore, further research is essential.

## Figures and Tables

**Table 1:** Content of selected oxides in input raw materials. (FA—fly ashes, LOI—loss on ignition).

Oxide	Content [%]		
	FA	GGBS	CBPD
SiO_2_	50.89	33.81	5.32
Al_2_O_3_	21.34	8.14	1.46
Fe_2_O_3_	9.49	0.32	1.22
CaO	4.48	46.16	34.08
SO_3_	0.58	1.46	5.16
K_2_O	3.14	0.42	18.62
MgO	1.67	7.86	0.48
LOI	6.27	0.00	21.90

**Table 2:** Cement by-pass dust composition (in wt.%) measured by XRD.

Participant	Content [%]
Sylvite (KCl)	21.9
Free CaO	22.4
Portlandite (Ca(OH)_2_)	15.0
Arcanite (K_2_(SO)_4_)	14.4
Quartz (SiO_2_)	4.02
Larnite/Belite (Ca_2_SiO_4_)	19.2
Calcite (CaCO_3_)	1.36
Dolomite (CaMg(CO_3_)_2)_	1.67

**Table 3:** Composition of Na_2_SiO_3_ [33].

A	MJ	Value
SiO_2_ content	%	min. 44
pH	-	12.5
Molar weight	kg/mol	122.06
Relative density	g/cm^3^	2.6

**Table 4:** Composition of mixtures is given in [g].

Mat.	GGBS	FA	CBPD	A	Water	Sand
REC 1	315	67.5	67.5	89.00	215	1350
REC 2	315	67.5	67.5	75.65	215	1350
REC 3	315	67.5	67.5	62.30	215	1350
REF 1	450	x	x	89.00	215	1350
REF 2	450	x	x	75.65	215	1350
REF 3	450	x	x	62.30	215	1350

## Data Availability

Not applicable.
